# Bromidotricarbon­yl[2-phenyl-5-(pyridin-2-yl-κ*N*)-1,3,4-oxadiazole-κ*N* ^4^]rhenium(I) dichloro­methane monosolvate

**DOI:** 10.1107/S1600536810052360

**Published:** 2010-12-18

**Authors:** Lin-Fang Shi, Zhen-Jun Si, Yan-Wei Li, Hua-Ru Cao, Ying Guan

**Affiliations:** a College of Sciences, Zhejiang A&F University, Lin’an, Hangzhou, Zhejiang 311300, People’s Republic of China; b School of Materials Science and Engineering, Changchun University of Science and Technology, Changchun 130000, People’s Republic of China; c School of Chemical Engineering & Technology, Harbin Institute of Technology, Harbin 150001, People’s Republic of China

## Abstract

In the title rhenium(I) complex, [ReBr(C_13_H_9_N_3_O)(CO)_3_]·CH_2_Cl_2_, the dichloro­methane solvent mol­ecule is disordered over two positions with an occupancy ratio of 0.81 (15):0.19 (15). The Re^I^ atom is coordinated by two N atoms from a 2-phenyl-5-(pyridin-2-yl-κ*N*)-1,3,4-oxadiazole (*L*) ligand, three C atoms from three carbonyl groups and a Br atom in a distorted octa­hedral geometry. The three rings in *L* are almost coplanar (a mean plane fitted through all non-H atoms of this ligand has an r.m.s. deviation of 0.063 Å), and the carbonyl ligands are coordinated in a *fac* arrangement.

## Related literature

For background to organic light emitting diodes, see: Li *et al.* (2005 ▶); Wong *et al.* (2005 ▶). For phospho­rescent materials, see: Kim *et al.* (2006 ▶); Lee *et al.* (2005 ▶); Bernhard *et al.* (2002 ▶). For the use of Re^I^ complexes as phospho­rescent materials, see: Gong *et al.* (1998 ▶); Li *et al.* (2001 ▶); Rajendran *et al.* (2000 ▶); Zhang *et al.* (2009 ▶). For the synthetic procecure, see: Demko & Sharpless (2001 ▶). 
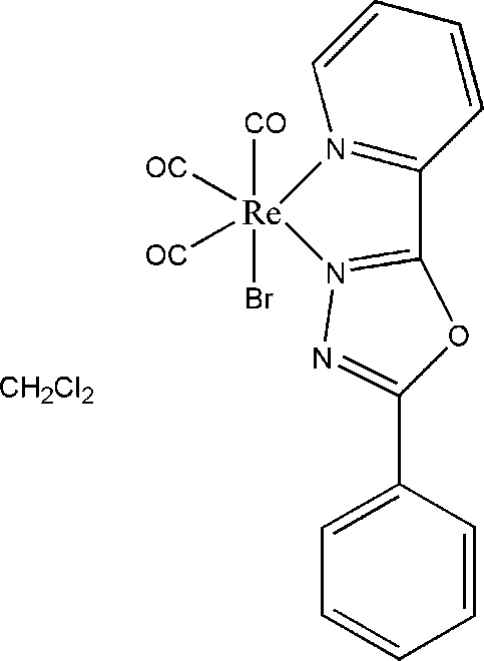


## Experimental

### 

#### Crystal data

[ReBr(C_13_H_9_N_3_O)(CO)_3_]·CH_2_Cl_2_
*M* *_r_* = 658.30
Monoclinic, 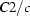

*a* = 12.492 (3) Å
*b* = 19.513 (4) Å
*c* = 16.835 (3) Å
β = 99.45 (3)°
*V* = 4047.9 (15) Å^3^
*Z* = 8
Mo *K*α radiation
μ = 8.27 mm^−1^
*T* = 293 K
0.20 × 0.16 × 0.11 mm

#### Data collection

Bruker SMART CCD area-detector diffractometer
Absorption correction: multi-scan (*SADABS*; Bruker, 2001 ▶) *T*_min_ = 0.229, *T*_max_ = 0.409
19615 measured reflections
4631 independent reflections
3992 reflections with *I* > 2σ(*I*)
*R*_int_ = 0.104

#### Refinement

*R*[*F* ^2^ > 2σ(*F* ^2^)] = 0.042
*wR*(*F* ^2^) = 0.107
*S* = 1.07
4631 reflections
276 parameters
36 restraints
H-atom parameters constrained
Δρ_max_ = 2.88 e Å^−3^
Δρ_min_ = −1.44 e Å^−3^

### 

Data collection: *SMART* (Bruker, 2007 ▶); cell refinement: *SAINT* (Bruker, 2007 ▶); data reduction: *SAINT*; program(s) used to solve structure: *SHELXS97* (Sheldrick, 2008 ▶); program(s) used to refine structure: *SHELXL97* (Sheldrick, 2008 ▶); molecular graphics: *SHELXTL* (Sheldrick, 2008 ▶); software used to prepare material for publication: *SHELXTL*.

## Supplementary Material

Crystal structure: contains datablocks global, I. DOI: 10.1107/S1600536810052360/nk2078sup1.cif

Structure factors: contains datablocks I. DOI: 10.1107/S1600536810052360/nk2078Isup2.hkl

Additional supplementary materials:  crystallographic information; 3D view; checkCIF report

## Figures and Tables

**Table 1:** Selected bond lengths (Å)

Re1—C1	1.884 (7)
Re1—C3	1.893 (7)
Re1—C2	1.920 (7)
Re1—N2	2.173 (4)
Re1—N1	2.228 (4)
Re1—Br1	2.6228 (11)

**Fig. 1.**
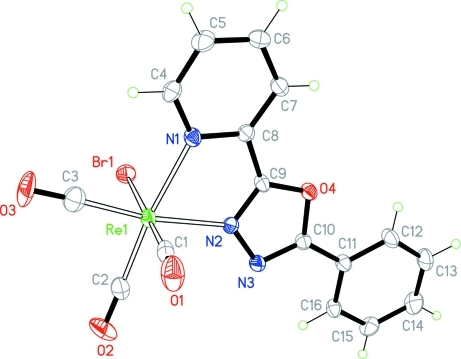
The asymmetric unit of [Re(CO)3(L)Br].CH2Cl2, displacement ellipsoids are drawn at the 30% probability level. The disordered dichloromethane molecule is omitted. (arbitrary spheres for the H atoms).

## supplementary crystallographic information

### Comment

Interest in next generation displays and lighting technologies has stimulated research on organic light-emitting materials (Li *et al.*, 2005; Wong *et al.*, 2005), especially phosphorescent materials (Kim *et al.*, 2006; Lee *et al.*, 2005). As a result, transition metal phosphorescent complexes (Bernhard *et al.*, 2002) have been studied intensively throughout the world. In order to further explore novel phosphorescent materials, several researchers paid attention to Re^I^ complexes (Gong *et al.*, 1998; Li *et al.*, 2001), of which the d^6^ electronic configuration is identical to that of the corresponding Os(II) and Ir(III) systems. Therefore, it is pressing to explore new Re^I^ complexes served as luminescent materials. In this article, we report the successful synthesis of a novel Re^I^ complex which contains the oxadiazole ligand of 2-phenyl-5-(pyridin-2-yl)-1,3,4-oxadiazole, and characterized its structure by single-crystal X-ray diffraction analysis. Its luminescent property will be further studied in the coming research.

The structure of complex [Re(CO)_3_(*L*)Br].CH_2_Cl_2_, is shown in Figure 1. One molecule of solvent dichloromethane is present in the asymmetric unit. This was refined as disordered over two positions, with occupancies of 0.81 (15):0.19 (15).. The coordination geometry at the Re atom is a distorted octahedron with the three CO ligands arranged in a *fac*-fashion. The distances of C(1), C(2), and C(3) to Re(1) are 1.884 (7), 1.920 (7), and 1.893 (7)Å, respectively, and the Re—N bonds distances are 2.228 (4) and 2.173 (4)Å. The CO ligands are linearly coordinated for the bond angles of O—C—Re are 174.8 (6), 177.5 (7) and 177.4 (7)°, respectively, which are close to 180°. Furthermore, the bond angles between adjacent CO carbon atoms are 87.9 (3), 89.1 (3) and 89.8 (3)°, respectively, which are close to 90°, but the bond angle between the coordinated nitrogen atoms of ligand is 73.71 (17)°, which is much less than 90°. All other bond distances and angles are comparable to those found for the related Re^I^ complexes (Rajendran *et al.*, 2000).

Furthermore, a kind of inter-molecular face-to-face stacking present in the molecular structure of [Re(CO)_3_(*L*)Br].CH_2_Cl_2_: the 1,3,4-oxadiazole moiety in one molecule is almost parallel to the other one from another complex , and the approximate distance between the two closest atoms (N2—N3) is only 3.376°. Thus a bonded dual-molecule structure is constructed in the complex molecule which is believed a rigid one and will prevent geometric relaxation effectively (Zhang *et al.*, 2009). Such rigid structure is promised possessing excellent luminescent properties.

### Experimental

The oxadiazole ligand was synthesized as follows: 5-(2-pyridyl)tetrazole (Demko *et al.*, 2001)(1.48 g, 10 mmol), pyridine (25 ml) and benzoyl chloride (1.41 g, 10 mmol) were added to a 50 ml round bottom flask and refluxed for 72 h. The crude product was then purified by column chromatography. Yield 1.61 g (59.6%). IR (KBr pellet): 3055 (*w*), 1616 (*w*), 1547 (*s*), 1481 (*s*), 1452 (*vs*), 1387 (*s*), 1146 (*m*), 1072 (*m*), 1024 (w), 793 (*s*), 715 (*vs*) cm-1. [Re(CO)_3_(*L*)Br] was synthesized according to the following procedure: *L* (0.05 g, 0.210 mmol) and Re(CO)_5_Br (0.08 g, 0.200 mmol) were refluxed in 15 ml of toluene for 6 h. After the mixture was cooled to RT, the solvent was removed in a water bath under reduced pressure. The resulting yellow solid was purified by silica gel column chromatography with acetic acid ethyl ester and dichloromethane (*v*/*v* = 10:1). Yellow single crystals of complexes 2 suitable for X-ray diffraction studies were grown from slow evaporation of a CH_2_Cl_2_ solution.

### Refinement

One molecule of solvent dichloromethane is present in the asymmetric unit. This was refined as disordered over two positions, with occupancies of 0.81 (15):0.19 (15). H atoms were identified from a difference map and refined using U_iso_(H) = 1.2U_eq_(C) and constrained C-H distances.

### Crystal data

**Table:**

[ReBr(C_13_H_9_N_3_O)(CO)_3_]·CH_2_Cl_2_	*F*(000) = 2480
*M**_r_* = 658.30	*D*_x_ = 2.160 Mg m^−^^3^
Monoclinic, *C*2/*c*	Mo *K*α radiation, λ = 0.71073 Å
Hall symbol: -C 2yc	Cell parameters from 4631 reflections
*a* = 12.492 (3) Å	θ = 2.3–27.5°
*b* = 19.513 (4) Å	µ = 8.27 mm^−^^1^
*c* = 16.835 (3) Å	*T* = 293 K
β = 99.45 (3)°	Block, yellow
*V* = 4047.9 (15) Å^3^	0.20 × 0.16 × 0.11 mm
*Z* = 8	

### Data collection

**Table:**

Bruker SMART CCD area-detector diffractometer	4631 independent reflections
Radiation source: fine-focus sealed tube	3992 reflections with *I* > 2σ(*I*)
graphite	*R*_int_ = 0.104
ω scans	θ_max_ = 27.5°, θ_min_ = 3.2°
Absorption correction: multi-scan (*SADABS*; Bruker, 2001)	*h* = −16→15
*T*_min_ = 0.229, *T*_max_ = 0.409	*k* = −25→25
19615 measured reflections	*l* = −21→21

### Refinement

**Table:**

Refinement on *F*^2^	Secondary atom site location: difference Fourier map
Least-squares matrix: full	Hydrogen site location: inferred from neighbouring sites
*R*[*F*^2^ > 2σ(*F*^2^)] = 0.042	H-atom parameters constrained
*wR*(*F*^2^) = 0.107	*w* = 1/[σ^2^(*F*_o_^2^) + (0.0507*P*)^2^ + 6.9046*P*] where *P* = (*F*_o_^2^ + 2*F*_c_^2^)/3
*S* = 1.07	(Δ/σ)_max_ = 0.002
4631 reflections	Δρ_max_ = 2.88 e Å^−^^3^
276 parameters	Δρ_min_ = −1.44 e Å^−^^3^
36 restraints	Extinction correction: *SHELXS97*(Sheldrick, 2008), Fc^*^=kFc[1+0.001xFc^2^λ^3^/sin(2θ)]^-1/4^
Primary atom site location: structure-invariant direct methods	Extinction coefficient: 0.00089 (8)

### Special details

**Table:**

Geometry. All e.s.d.'s (except the e.s.d. in the dihedral angle between two l.s. planes) are estimated using the full covariance matrix. The cell e.s.d.'s are taken into account individually in the estimation of e.s.d.'s in distances, angles and torsion angles; correlations between e.s.d.'s in cell parameters are only used when they are defined by crystal symmetry. An approximate (isotropic) treatment of cell e.s.d.'s is used for estimating e.s.d.'s involving l.s. planes.
Refinement. Refinement of *F*^2^ against ALL reflections. The weighted *R*-factor *wR* and goodness of fit *S* are based on *F*^2^, conventional *R*-factors *R* are based on *F*, with *F* set to zero for negative *F*^2^. The threshold expression of *F*^2^ > σ(*F*^2^) is used only for calculating *R*-factors(gt) *etc*. and is not relevant to the choice of reflections for refinement. *R*-factors based on *F*^2^ are statistically about twice as large as those based on *F*, and *R*- factors based on ALL data will be even larger.

### Fractional atomic coordinates and isotropic or equivalent isotropic displacement parameters (Å^2^)

**Table:**

	*x*	*y*	*z*	*U*_iso_*/*U*_eq_	Occ. (<1)
Re1	0.763878 (16)	0.394333 (9)	0.240070 (14)	0.03250 (12)	
Br1	0.62778 (5)	0.40803 (3)	0.10476 (4)	0.04074 (16)	
O1	0.9100 (4)	0.3751 (3)	0.4011 (3)	0.0696 (16)	
O2	0.8889 (5)	0.2798 (3)	0.1726 (4)	0.0808 (18)	
O3	0.6198 (4)	0.2847 (3)	0.2967 (4)	0.0825 (19)	
O4	0.8670 (3)	0.59195 (17)	0.1890 (3)	0.0314 (8)	
N1	0.6852 (3)	0.4871 (2)	0.2817 (3)	0.0314 (9)	
N2	0.8475 (3)	0.4820 (2)	0.1993 (3)	0.0309 (9)	
N3	0.9313 (3)	0.4917 (2)	0.1559 (3)	0.0335 (10)	
C1	0.8579 (5)	0.3847 (3)	0.3391 (4)	0.0444 (15)	
C2	0.8427 (5)	0.3231 (3)	0.1961 (5)	0.0508 (16)	
C3	0.6735 (5)	0.3274 (4)	0.2764 (4)	0.0527 (16)	
C4	0.6067 (4)	0.4881 (3)	0.3268 (4)	0.0384 (12)	
H4	0.5784	0.4464	0.3403	0.046*	
C5	0.5655 (5)	0.5467 (3)	0.3545 (4)	0.0468 (14)	
H5	0.5123	0.5446	0.3871	0.056*	
C6	0.6049 (5)	0.6097 (3)	0.3328 (4)	0.0445 (15)	
H6	0.5779	0.6504	0.3505	0.053*	
C7	0.6850 (5)	0.6109 (3)	0.2844 (4)	0.0396 (14)	
H7	0.7113	0.6520	0.2673	0.047*	
C8	0.7239 (4)	0.5490 (3)	0.2628 (3)	0.0308 (11)	
C9	0.8113 (4)	0.5412 (2)	0.2168 (3)	0.0297 (10)	
C10	0.9406 (4)	0.5585 (2)	0.1511 (3)	0.0304 (10)	
C11	1.0160 (4)	0.5964 (2)	0.1110 (3)	0.0320 (11)	
C12	1.0231 (5)	0.6674 (3)	0.1184 (4)	0.0441 (14)	
H12	0.9795	0.6912	0.1487	0.053*	
C13	1.0960 (5)	0.7015 (3)	0.0798 (5)	0.0534 (16)	
H13	1.1024	0.7488	0.0849	0.064*	
C14	1.1604 (5)	0.6663 (4)	0.0334 (4)	0.0516 (16)	
H14	1.2089	0.6901	0.0073	0.062*	
C15	1.1523 (5)	0.5966 (3)	0.0260 (4)	0.0442 (15)	
H15	1.1948	0.5732	−0.0056	0.053*	
C16	1.0816 (4)	0.5609 (3)	0.0651 (3)	0.0342 (11)	
H16	1.0774	0.5134	0.0611	0.041*	
Cl1	0.6313 (10)	0.7067 (5)	0.0785 (8)	0.115 (3)	0.810 (15)
Cl2	0.6782 (3)	0.57172 (14)	0.0268 (2)	0.0650 (12)	0.810 (15)
C17	0.6743 (17)	0.6573 (6)	0.0037 (9)	0.102 (5)	0.810 (15)
H17A	0.7460	0.6722	−0.0037	0.123*	0.810 (15)
H17B	0.6254	0.6644	−0.0466	0.123*	0.810 (15)
Cl1'	0.666 (5)	0.7100 (17)	0.087 (3)	0.121 (16)	0.190 (15)
Cl2'	0.715 (4)	0.591 (3)	0.0034 (16)	0.201 (18)	0.190 (15)
C17'	0.660 (9)	0.681 (3)	0.017 (5)	0.102 (5)	0.19
H17C	0.6951	0.7113	−0.0162	0.123*	0.190 (15)
H17D	0.5835	0.6799	−0.0067	0.123*	0.190 (15)

### Atomic displacement parameters (Å^2^)

**Table:**

	*U*^11^	*U*^22^	*U*^33^	*U*^12^	*U*^13^	*U*^23^
Re1	0.03620 (16)	0.02314 (14)	0.0399 (2)	−0.00311 (6)	0.01139 (11)	0.00443 (7)
Br1	0.0422 (3)	0.0423 (3)	0.0395 (4)	0.0007 (2)	0.0119 (2)	−0.0024 (2)
O1	0.086 (3)	0.054 (3)	0.061 (4)	−0.020 (3)	−0.013 (3)	0.018 (3)
O2	0.089 (4)	0.043 (3)	0.115 (5)	0.024 (3)	0.032 (3)	−0.016 (3)
O3	0.079 (3)	0.072 (4)	0.100 (5)	−0.041 (3)	0.026 (3)	0.025 (3)
O4	0.0387 (18)	0.0246 (15)	0.034 (2)	−0.0016 (14)	0.0167 (16)	0.0007 (15)
N1	0.037 (2)	0.027 (2)	0.031 (2)	−0.0016 (16)	0.0096 (18)	0.0030 (18)
N2	0.0291 (19)	0.025 (2)	0.041 (3)	−0.0015 (16)	0.0141 (17)	0.0053 (18)
N3	0.033 (2)	0.026 (2)	0.043 (3)	0.0001 (16)	0.0143 (18)	0.0019 (19)
C1	0.046 (3)	0.028 (2)	0.058 (5)	−0.009 (2)	0.005 (3)	0.000 (3)
C2	0.055 (3)	0.035 (3)	0.065 (5)	−0.006 (3)	0.016 (3)	0.001 (3)
C3	0.054 (3)	0.055 (4)	0.050 (4)	−0.006 (3)	0.009 (3)	0.004 (3)
C4	0.038 (3)	0.042 (3)	0.037 (3)	−0.007 (2)	0.014 (2)	0.006 (2)
C5	0.046 (3)	0.056 (4)	0.042 (4)	−0.003 (3)	0.021 (3)	−0.003 (3)
C6	0.045 (3)	0.043 (3)	0.049 (4)	0.003 (2)	0.019 (3)	−0.009 (3)
C7	0.045 (3)	0.029 (3)	0.047 (4)	−0.001 (2)	0.015 (3)	−0.004 (2)
C8	0.032 (2)	0.029 (2)	0.033 (3)	−0.0020 (18)	0.011 (2)	0.0009 (19)
C9	0.038 (2)	0.022 (2)	0.030 (3)	−0.0014 (18)	0.009 (2)	0.0001 (19)
C10	0.033 (2)	0.029 (2)	0.031 (3)	−0.0012 (19)	0.008 (2)	−0.001 (2)
C11	0.039 (3)	0.028 (2)	0.032 (3)	−0.0015 (19)	0.012 (2)	0.001 (2)
C12	0.055 (3)	0.025 (2)	0.057 (4)	−0.001 (2)	0.023 (3)	0.004 (2)
C13	0.060 (4)	0.031 (3)	0.072 (5)	−0.013 (3)	0.018 (3)	0.004 (3)
C14	0.057 (3)	0.053 (4)	0.049 (4)	−0.014 (3)	0.021 (3)	0.010 (3)
C15	0.041 (3)	0.057 (4)	0.039 (4)	−0.005 (3)	0.020 (3)	−0.005 (3)
C16	0.043 (3)	0.030 (2)	0.032 (3)	−0.006 (2)	0.012 (2)	−0.004 (2)
Cl1	0.143 (5)	0.073 (4)	0.129 (7)	0.003 (3)	0.029 (5)	−0.024 (3)
Cl2	0.087 (2)	0.0521 (17)	0.053 (2)	−0.0020 (11)	0.0019 (14)	−0.0019 (11)
C17	0.209 (15)	0.038 (7)	0.074 (9)	0.007 (9)	0.064 (9)	0.010 (6)
Cl1'	0.24 (5)	0.034 (8)	0.084 (13)	−0.008 (15)	−0.01 (2)	−0.005 (8)
Cl2'	0.24 (3)	0.32 (4)	0.044 (12)	0.11 (3)	0.014 (16)	−0.036 (18)
C17'	0.209 (15)	0.038 (7)	0.074 (9)	0.007 (9)	0.064 (9)	0.010 (6)

### Geometric parameters (Å, °)

**Table:**

Re1—C1	1.884 (7)	C7—H7	0.9300
Re1—C3	1.893 (7)	C8—C9	1.446 (7)
Re1—C2	1.920 (7)	C10—C11	1.448 (7)
Re1—N2	2.173 (4)	C11—C12	1.391 (7)
Re1—N1	2.228 (4)	C11—C16	1.399 (7)
Re1—Br1	2.6228 (11)	C12—C13	1.374 (9)
O1—C1	1.152 (8)	C12—H12	0.9300
O2—C2	1.132 (8)	C13—C14	1.392 (10)
O3—C3	1.157 (8)	C13—H13	0.9300
O4—C9	1.337 (6)	C14—C15	1.368 (9)
O4—C10	1.369 (6)	C14—H14	0.9300
N1—C4	1.335 (7)	C15—C16	1.375 (8)
N1—C8	1.357 (6)	C15—H15	0.9300
N2—C9	1.293 (6)	C16—H16	0.9300
N2—N3	1.385 (6)	Cl1—C17	1.739 (19)
N3—C10	1.314 (6)	Cl2—C17	1.713 (12)
C4—C5	1.367 (9)	C17—H17A	0.9700
C4—H4	0.9300	C17—H17B	0.9700
C5—C6	1.395 (8)	Cl1'—C17'	1.30 (9)
C5—H5	0.9300	Cl2'—C17'	1.90 (6)
C6—C7	1.391 (9)	C17'—H17C	0.9700
C6—H6	0.9300	C17'—H17D	0.9700
C7—C8	1.373 (7)		
			
C1—Re1—C3	87.9 (3)	N1—C8—C9	111.2 (4)
C1—Re1—C2	89.1 (3)	C7—C8—C9	124.4 (5)
C3—Re1—C2	89.8 (3)	N2—C9—O4	111.2 (4)
C1—Re1—N2	95.5 (2)	N2—C9—C8	122.6 (4)
C3—Re1—N2	171.3 (2)	O4—C9—C8	126.2 (4)
C2—Re1—N2	98.3 (2)	N3—C10—O4	111.8 (4)
C1—Re1—N1	92.7 (2)	N3—C10—C11	127.4 (5)
C3—Re1—N1	98.1 (3)	O4—C10—C11	120.8 (4)
C2—Re1—N1	171.9 (2)	C12—C11—C16	120.5 (5)
N2—Re1—N1	73.71 (17)	C12—C11—C10	120.3 (5)
C1—Re1—Br1	178.2 (2)	C16—C11—C10	119.2 (4)
C3—Re1—Br1	90.9 (2)	C13—C12—C11	118.6 (6)
C2—Re1—Br1	92.2 (2)	C13—C12—H12	120.7
N2—Re1—Br1	85.55 (12)	C11—C12—H12	120.7
N1—Re1—Br1	86.19 (11)	C12—C13—C14	121.0 (6)
C9—O4—C10	103.8 (4)	C12—C13—H13	119.5
C4—N1—C8	116.3 (5)	C14—C13—H13	119.5
C4—N1—Re1	126.4 (4)	C15—C14—C13	120.0 (6)
C8—N1—Re1	117.2 (3)	C15—C14—H14	120.0
C9—N2—N3	108.7 (4)	C13—C14—H14	120.0
C9—N2—Re1	115.3 (3)	C14—C15—C16	120.3 (6)
N3—N2—Re1	135.9 (3)	C14—C15—H15	119.9
C10—N3—N2	104.5 (4)	C16—C15—H15	119.9
O1—C1—Re1	174.8 (6)	C15—C16—C11	119.6 (5)
O2—C2—Re1	177.5 (7)	C15—C16—H16	120.2
O3—C3—Re1	177.4 (7)	C11—C16—H16	120.2
N1—C4—C5	123.9 (5)	Cl1—C17—Cl2	112.2 (9)
N1—C4—H4	118.0	Cl1—C17—H17A	109.2
C5—C4—H4	118.0	Cl2—C17—H17A	109.2
C4—C5—C6	118.7 (6)	Cl1—C17—H17B	109.2
C4—C5—H5	120.6	Cl2—C17—H17B	109.2
C6—C5—H5	120.6	H17A—C17—H17B	107.9
C7—C6—C5	119.1 (5)	Cl1'—C17'—Cl2'	123 (5)
C7—C6—H6	120.5	Cl1'—C17'—H17C	106.7
C5—C6—H6	120.5	Cl2'—C17'—H17C	106.7
C8—C7—C6	117.4 (5)	Cl1'—C17'—H17D	106.7
C8—C7—H7	121.3	Cl2'—C17'—H17D	106.7
C6—C7—H7	121.3	H17C—C17'—H17D	106.6
N1—C8—C7	124.4 (5)		
			
C1—Re1—N1—C4	−81.4 (5)	C8—N1—C4—C5	−0.4 (8)
C3—Re1—N1—C4	6.9 (5)	Re1—N1—C4—C5	176.1 (4)
C2—Re1—N1—C4	176.2 (15)	N1—C4—C5—C6	1.9 (10)
N2—Re1—N1—C4	−176.3 (5)	C4—C5—C6—C7	−0.5 (10)
Br1—Re1—N1—C4	97.2 (4)	C5—C6—C7—C8	−2.2 (10)
C1—Re1—N1—C8	95.2 (4)	C4—N1—C8—C7	−2.6 (8)
C3—Re1—N1—C8	−176.6 (4)	Re1—N1—C8—C7	−179.5 (4)
C2—Re1—N1—C8	−7.3 (18)	C4—N1—C8—C9	177.6 (5)
N2—Re1—N1—C8	0.2 (3)	Re1—N1—C8—C9	0.7 (6)
Br1—Re1—N1—C8	−86.3 (4)	C6—C7—C8—N1	3.9 (9)
C1—Re1—N2—C9	−92.4 (4)	C6—C7—C8—C9	−176.3 (6)
C3—Re1—N2—C9	20.2 (18)	N3—N2—C9—O4	−1.1 (6)
C2—Re1—N2—C9	177.7 (4)	Re1—N2—C9—O4	−179.6 (3)
N1—Re1—N2—C9	−1.2 (4)	N3—N2—C9—C8	−179.4 (5)
Br1—Re1—N2—C9	86.1 (4)	Re1—N2—C9—C8	2.2 (7)
C1—Re1—N2—N3	89.7 (5)	C10—O4—C9—N2	1.0 (6)
C3—Re1—N2—N3	−157.6 (15)	C10—O4—C9—C8	179.2 (5)
C2—Re1—N2—N3	−0.2 (6)	N1—C8—C9—N2	−1.9 (8)
N1—Re1—N2—N3	−179.1 (5)	C7—C8—C9—N2	178.3 (5)
Br1—Re1—N2—N3	−91.8 (5)	N1—C8—C9—O4	−179.9 (4)
C9—N2—N3—C10	0.8 (6)	C7—C8—C9—O4	0.3 (9)
Re1—N2—N3—C10	178.7 (4)	N2—N3—C10—O4	−0.1 (6)
C3—Re1—C1—O1	0(7)	N2—N3—C10—C11	−179.4 (5)
C2—Re1—C1—O1	−90 (7)	C9—O4—C10—N3	−0.5 (6)
N2—Re1—C1—O1	172 (7)	C9—O4—C10—C11	178.8 (5)
N1—Re1—C1—O1	98 (7)	N3—C10—C11—C12	−173.4 (6)
Br1—Re1—C1—O1	47 (11)	O4—C10—C11—C12	7.4 (8)
C1—Re1—C2—O2	47 (14)	N3—C10—C11—C16	6.4 (8)
C3—Re1—C2—O2	−41 (14)	O4—C10—C11—C16	−172.8 (5)
N2—Re1—C2—O2	142 (14)	C16—C11—C12—C13	−0.1 (9)
N1—Re1—C2—O2	150 (13)	C10—C11—C12—C13	179.6 (6)
Br1—Re1—C2—O2	−132 (14)	C11—C12—C13—C14	0.9 (10)
C1—Re1—C3—O3	−103 (14)	C12—C13—C14—C15	−0.5 (11)
C2—Re1—C3—O3	−13 (14)	C13—C14—C15—C16	−0.7 (10)
N2—Re1—C3—O3	144 (13)	C14—C15—C16—C11	1.5 (9)
N1—Re1—C3—O3	165 (14)	C12—C11—C16—C15	−1.1 (8)
Br1—Re1—C3—O3	79 (14)	C10—C11—C16—C15	179.1 (5)
